# Upregulation of α-ENaC induces pancreatic β-cell dysfunction, ER stress, and SIRT2 degradation

**DOI:** 10.7555/JBR.37.20230128

**Published:** 2024-05-21

**Authors:** Xue Zhang, Dan Zhang, Lei Huo, Xin Zhou, Jia Zhang, Min Li, Dongming Su, Peng Sun, Fang Chen, Xiubin Liang

**Affiliations:** 1 Department of Pathophysiology, Nanjing Medical University, Nanjing, Jiangsu 211166, China; 2 Department of Pathology, Nanjing Drum Tower Hospital, the Affiliated Hospital of Nanjing University Medical School, Nanjing, Jiangsu 210009, China; 3 Key Laboratory of Human Functional Genomics of Jiangsu Province, Department of Biochemistry and Molecular Biology, Nanjing Medical University, Nanjing, Jiangsu 211166, China; 4 Department of Pathology, Nanjing Medical University, Nanjing, Jiangsu 211166, China

**Keywords:** α-ENaC, pancreatic β-cells, type 2 diabetes mellitus, endoplasmic reticulum stress, sirtuin 2

## Abstract

Islet beta cells (β-cells) produce insulin in response to high blood glucose levels, which is essential for preserving glucose homeostasis. Voltage-gated ion channels in β-cells, including Na^+^, K^+^, and Ca^2+^ channels, aid in the release of insulin. The epithelial sodium channel alpha subunit (α-ENaC), a voltage-independent sodium ion channel, is also expressed in human pancreatic endocrine cells. However, there is no reported study on the function of ENaC in the β-cells. In the current study, we found that α-ENaC was expressed in human pancreatic glandule and pancreatic islet β-cells. In the pancreas of *db/db* mice and high-fat diet-induced mice, and in mouse islet β-cells (MIN6 cells) treated with palmitate, α-ENaC expression was increased. When α-ENaC was overexpressed in MIN6 cells, insulin content and glucose-induced insulin secretion were significantly reduced. On the other hand, palmitate injured islet β-cells and suppressed insulin synthesis and secretion, but increased α-ENaC expression in MIN6 cells. However, α-ENaC knockout (*Scnn1a*^−/−^) in MIN6 cells attenuated β-cell disorder induced by palmitate. Furthermore, α-ENaC regulated the ubiquitylation and degradation of sirtuin 2 in β-cells. α-ENaC also modulated β-cell function in correlation with the inositol-requiring enzyme 1 alpha/X-box binding protein 1 (IRE1α/XBP1) and protein kinase RNA-like endoplasmic reticulum kinase/C/EBP homologous protein (PERK/CHOP) endoplasmic reticulum stress pathways. These results suggest that α-ENaC may play a novel role in insulin synthesis and secretion in the β-cells, and the upregulation of α-ENaC promotes islet β-cell dysfunction. In conclusion, α-ENaC may be a key regulator involved in islet β-cell damage and a potential therapeutic target for type 2 diabetes mellitus.

## Introduction

The signs and symptoms of type 2 diabetes mellitus (T2DM) include insulin resistance and a progressive loss of pancreatic β-cell mass and function^[1–2]^. The damage to islet β-cells leads to disorders of insulin synthesis and secretion, and further affects glucose homeostasis in the body. A long-term exposure to high levels of saturated fatty acids (*e.g.*, palmitic acid and stearic acid) is thought to be one of the main causes of this damage^[3–5]^. It is widely reported that lipid metabolism and dysregulated intracellular pathways, including oxidative stress, endoplasmic reticulum (ER) stress, and other cellular stress responses, are involved in the lipotoxic-induced cell death^[6–7]^, but the complete molecular mechanisms of fatty acid-induced β-cell apoptosis remain unclear^[8]^.

It is well known that electric activity through ion channels participates in β-cell depolarization for insulin secretion^[9]^. To date, at least six different ionic channels, including ATP-sensitive potassium channels (K_ATP_), transient receptor potential channels (TRPs), voltage-gated sodium channels (Na_v_), low- and high-voltage-activated calcium channels (Ca_v_), voltage-dependent potassium channels (K_v_), and calcium-sensitive voltage-dependent potassium channels, have been reported to be involved in insulin secretion of pancreatic beta cells in mammals^[10–12]^.

The human epithelial sodium channel (ENaC) is a voltage-independent sodium ion channel composed of four homologous subunits (*i.e.*, α, β, γ, and δ), of which the α subunit (α-ENaC) plays a major role in its functional activity^[13–14]^. The subunit δ is not expressed in rodents, and its functional role is unclear^[15]^. ENaC is mainly located in the epithelial tissues of the cortical collecting duct of the kidney and the airway for Na^+^ reabsorption there^[16]^. In the kidney, ENaC mediates apical Na^+^ reabsorption to regulate salt and water homeostasis, and thus extracellular volume and blood pressure^[17]^. ENaC mutations in epithelia contribute to the symptoms of hypertension in the Liddle syndrome and hypotension in pseudohypoaldosteronism type 1^[18]^. In the lungs, ENaC is also expressed in the epithelia, is associated with the function of cystic fibrosis transmembrane conductance regulator^[19]^, and plays a crucial role in maintaining lung alveolar fluid balance. In addition, ENaC subunits are widely expressed in extrapulmonary and extrarenal tissues throughout the body, including the colon, sweat glands, skin, blood vessels, and pancreas. Therefore, ENaC is implicated in a wide range of physiological roles, including taste perception^[20–21]^, cellular differentiation^[22–24]^, cell proliferation and apoptosis^[25–28]^, cell migration^[29]^, and other processes^[29–31]^. In 1994, human α-ENaC transcripts were reported in the pancreas by McDonald *et al*^[32]^, but no information about cellular localization of α-ENaC was provided. The Human Protein Atlas (https://www.proteinatlas.org) reveals that β-ENaC, γ-ENaC, and δ-ENaC are all present in extremely low levels in the pancreas, whereas α-ENaC is abundantly expressed in both human islet and pancreatic exocrine tissues. However, other electrophysiological experiments showed that despite the expression of ENaC in the stimulated and unstimulated pancreatic ducts, there was no evidence for its functionality in either ducts^[33–34]^. Therefore, physiological and pathophysiological roles of α-ENaC in the pancreas are unclear.

We previously showed that the α-ENaC gene knockout (*Scnn1a*^−/−^) led to an increase in cell viability and insulin content in MIN6 cells^[35]^. It is still unclear whether α-ENaC may play a role in the disorders of islet β-cells. In the current study, we focused on how ENaC affected β-cell function and how α-ENaC was involved in the development and progression of T2DM.

## Materials and methods

### Animals

Male C57BL/6 mice and *db/db* mice were obtained from the Model Animal Research Center of Nanjing University. Four-week-old male C57BL/6 mice (*n* = 3 for each group) underwent 12-week feeding with a high-fat diet (HFD; 60% fat, D12492, Research Diets) or a normal chow diet (NCD) with a 12/12 h light-dark cycle. All animal experiments were approved by the Institutional Review Board of Nanjing Medical University and performed following the guidelines established by the Animal Ethical and Welfare Committee of Nanjing Medical University (Approval No. IACUC-2007041).

### Isolation of pancreatic islets

Islets were isolated from eight-week-old male C57BL/6 mice and *db/db* mice fed with an NCD, and the isolation and incubation of islets were performed as described previously^[36]^. In brief, islets were first digested by injection of 1 mg/mL collagenase (Sigma-Aldrich, St. Louis, MO, USA) through the common bile duct, and then were washed and separated by gradient centrifugation in Histopaque (Sigma-Aldrich), and finally observed by stereomicroscopy and collected by pipette.

### Human sample collection

Five human pancreatic tissues (adjacent to carcinoma) embedded in paraffin were kindly gifted by Dr. Feng Zhu from Sir Run Run Hospital of Nanjing Medical University. None of the cases had a diagnosis of diabetes or hypertension, and the clinical parameters are presented in ***Supplementary Table 1*** (available online). Procedures involving human samples were approved by Sir Run Run Hospital of Nanjing Medical University (Approval No. 2019-SR-S031) and were consistent with the principles outlined in the Declaration of Helsinki.

### Reagents and antibodies

Palmitate (PA) and amiloride were obtained from Sigma-Aldrich and dissolved in absolute ethanol to obtain a 220 mmol/L stock solution that was used after complexing with bovine serum albumin (Sigma-Aldrich) solution in a 60 ℃ water bath. Amiloride was dissolved in dimethyl sulfoxide and stored at −20 ℃, diluted to the corresponding concentration, and used to pretreat mouse islet β-cells (MIN6) for 2 h before stimulation with PA. Thapsigargin (TG) was obtained from MedChemexpress (Monmouth Junction, NJ, USA) and dissolved in dimethyl sulfoxide.

Primary antibodies against α-ENaC (1∶1000 dilution; Cat. #SPC-403, StressMarq Biosciences, Victoria, British Columbia, Canada), sirtuin 2 (SIRT2; 1∶1000 dilution; Cat #19655-1-AP, ProteinTech Group, Rosemont, IL, USA), X-box binding protein 1 (XBP1; 1∶1000 dilution; Cat. #ab37152, Abcam, Cambridge, UK), and phosphorylated inositol-requiring enzyme 1 alpha (p-IRE1α; 1∶1000 dilution; Cat #ab48187, Abcam) were used in the experiments. Anti-poly ADP-ribose polymerase (PARP; Cat. #9542), anti-cleaved caspase3 (Cat. #9664S), anti-caspase3 (Cat. #9662S), anti-B-cell lymphoma-2 (BCL-2; Cat. #4223), anti-BCL2-associated X (BAX; Cat. #2772S), anti-AKT (Cat. #9272), anti-P-AKT (Cat. #9271), anti-BIP (Cat. #3177), anti-CHOP (Cat. #5554), anti-ubiquitin (Cat. #3933S), and anti-rabbit (Cat. #7074) were acquired from Cell Signaling Technology (Boston, MA, USA) and diluted 1∶1000 for Western blotting.

### Cell culture

Cell culture reagents were all purchased from Gibco (Grand Island, NY, USA). The MIIN6 cell line was obtained from the Division of Stem Cell Regulation Research (Osaka University Graduate School of Medicine, Suita, Osaka, Japan). MIN6 cells were maintained in DMEM (high glucose, without sodium pyruvate) supplemented with 15% fetal bovine serum and 50 μmol/L β-mercaptoethanol in a humidified atmosphere of 5% CO_2_ at 37 ℃, as previously described^[35,37]^.

### Plasmid constructs and transfection

The constructs of α-ENaC or SIRT2 plasmids were amplified from an ultimate openreading frame clone by PCR and cloned into the empty vector pcDNA3.1 (Addgene plasmid #138209, Addgene, Cambridge, MA, USA), as previously described^[37–38]^. All plasmid constructs were confirmed by DNA sequencing. MIN6 cells (1 × 10^6^ cells) were transfected with 5 μg of plasmids by the Lipofectamine 3000 Transfection Kit (Thermo Fisher Scientific, Waltham, MA, USA) according to the manufacturer's instructions.

### Cell proliferation and apoptosis assay

The proliferation and viability of the cells were detected by the Cell Counting Kit-8 (CCK-8) assay. Cells were grown in 96-well plates with the relevant treatment, and the plates were incubated for 2 h, 4 h, and 6 h after the addition of the CCK-8 assay reagent (10 μL per well; APExBIO, Houston, TX, USA). The optical density at 450 nm (OD450) was measured by Varioskan LUX microplate reader (Thermo Fisher Scientific). Cell apoptosis was measured by TdT-mediated dUTP nick end labeling (TUNEL) assay (Beyotime Biotechnology, Shanghai, China). Briefly, MIN6 and α-ENaC knockout (*Scnn1a*^−/−^) MIN6 cells were planted in six-well plates and fixed in a 4 ℃ formaldehyde solution for 24 h after different treatments, and then immersed in phosphate-buffered saline (PBS) containing 0.3% (v/v) Triton X-100 and incubated for 1 h with the configured TUNEL stain at 37 ℃. After washing three times with PBS, 4′,6-Diamidino-2-phenylindole dihydrochloride was added, and after sealing, the tablet was observed using a display microscope to evaluate apoptotic cells.

### Western blotting assay

Lysates obtained from MIN6 cells were resolved by 8% or 12% SDS-PAGE on gels and transferred to PVDF membranes (Millipore, Billerica, MA, USA). The unbound sites were blocked with 5% skim milk in Tris-buffered saline (TBS) for 2 h at room temperature. The blotted membranes were incubated with the primary antibodies at appropriate dilutions, then incubated with horseradish peroxidase-conjugated secondary antibodies, and finally subjected to enhanced chemiluminescence imaging (Thermo Fisher Scientific). Quantitative analysis of the Western blotting data was performed using Image Lab software (Bio-Rad, Hercules, CA, USA) and normalized to the expression of β-actin.

### Glucose-stimulated insulin secretion assay

MIN6 cells (2 × 10^6^ cells/well) were seeded in 48-well plates for the glucose-stimulated insulin secretion assay, followed by PBS rinsing and subsequent incubation at 37 ℃ in Krebs-Ringer bicarbonate buffer (115 mmol/L NaCl, 24 mmol/L NaHCO_3_, 5 mmol/L KCl, 2.5 mmol/L CaCl_2_, 1 mmol/L MgCl_2_, 10 mmol/L HEPES, and 0.2% BSA, pH 7.2) without glucose for 1 h. Next, the cells were incubated in Krebs-Ringer bicarbonate buffer containing 2 mmol/L or 16 mmol/L glucose for 1 h. After incubation, the supernatant was collected, and the insulin concentration was measured with a Mouse Insulin ELISA Kit (Crystal Chem, IL, USA).

### Real-time reverse transcriptase PCR (RT-qPCR)

The TRIzol reagent (Invitrogen, Carlsbad, CA, USA) was used for total RNA extraction. The SYBR Green Master Mix (Applied Biosystems, Foster, MI, USA) and real-time PCR system (Applied Biosystems Step OnePlus™, San Francisco, CA, USA) were performed for PCR. The relative expression levels of mRNAs were detected by RT-qPCR using the following primers: *Scnn1a* (α-ENaC), forward, 5′-AAGGGGGACAAGCGTGAAG-3′ and reverse, 5′-GCCAGTACATCATGCCGAAG-3′; *Pdx1*, forward, 5′-AGGAAAACAAGAGGACCCGT-3′ and reverse, 5′-CTTCATGCGACGGTTTTGGA-3′; *Mafa*, forward, 5′-AGGAGGAGGTCATCCGACTG-3′ and reverse, 5′-CTTCTCGCTCTCCAGAATGTG-3′; *Neurod1*, forward, 5′-ATGACCAAATCATACAGCGAGAG-3′ and reverse, 5′-TCTGCCTCGTGTTCCTCGT-3′; *Actb* (β-actin), forward, 5′-AGGCCAACCGTGAAAAGATG-3′ and reverse, 5′-AGAGCATAGCCCTCGTAGATGG-3′. The mRNA expression levels were calculated by the 2^ΔΔCT^ method and normalized to the housekeeping gene β-actin.

### Hematoxylin and eosin (HE) and immunohistochemistry (IHC)

Mouse pancreases were dissected, fixed with 4% paraformaldehyde in PBS, and embedded in paraffin. The HE stainings were performed to confirm the normal morphology in human pancreas samples. For IHC staining, the pancreas sections were placed in citrate buffer (pH 6.0) at 95 ℃ for 2 min for antigen repair, then incubated with the primary antibody against α-ENaC at 4 ℃ overnight, and stained with anti-rabbit IgG (Cat. #PV-6000; ZSGB-BIO, Beijing, China) the next day. DAB kits (ZSGB-BIO) were used to show the IHC staining and the nuclei were stained with hematoxylin. All images were captured by using a digital microscope (IX51 + DP72; OLYMPUS, Tokyo, Japan).

### Immunofluorescence (IF) staining

For IF staining, antigen retrieval was performed by boiling slides in 10 mmol/L citric acid buffer at 95 ℃ for 10 min. Endogenous peroxidases were quenched with 3% hydrogen peroxide for 10 min at room temperature, followed by incubation with mouse anti-insulin antibody (1∶500 dilution, Cat#8138S, Cell Signaling Technology) and rabbit anti-α-ENaC antibody at 4 ℃ overnight. After three washes with PBS, samples were incubated for 1 h at room temperature in secondary antibodies in a light-deprived humid chamber. Secondary antibodies were goat anti-mouse and goat anti-rabbit IgG coupled with Alexa 568 (red) or Alexa 488 (green) fluorochromes (1∶1000 dilution, Thermo Fisher Scientific), and samples were mounted with Fluoroshield containing DAPI (Sigma-Aldrich). All images were obtained using an Olympus confocal microscope processed using Olympus FV1000 software and analyzed with Image-Pro Plus software (Media Cybernetics, MD, USA).

### Cycloheximide (CHX) chase assay

The half-life of the SIRT2 protein was examined by the cycloheximide (CHX, Sigma-Aldrich) chase assay. Cells were treated with 100 μg/mL of CHX, which is a protein synthesis inhibitor, and then harvested at specific time points (0, 2, 4, and 8 h). Cell lysates were analyzed by Western blotting as described above.

### Immunoprecipitation (IP) assay

For IP assays, total cellular proteins were lysed in NP40 with 10 mmol/L Tris-HCl and 10 mmol/L NaCl. Protein concentrations were measured by the Coomassie brilliant blue G-250 assay, and 1 mg of protein was immunoprecipitated using a target antibody at 4 ℃ overnight and then incubated with 100 µL of protein A beads (Thermo Fisher Scientific) for another 2 to 6 h. Subsequently, the immunocomplexes were suspended with 2× SDS loading buffer and identified by Western blotting.

### Ubiquitination assay

To determine the ubiquitination of SIRT2, cells were treated with 20 µmol/L MG132, a proteasome inhibitor, for 8 h before the protein extraction and detection. The protein lysates were then incubated with an anti-SIRT2 antibody overnight, and mixed with protein A beads at 4 ℃ for 6 h. The released proteins were finally subjected to Western blotting using a 1∶1000 dilution of an anti-ubiquitin antibody.

### Statistical analysis

In the current study, all experiments were repeated at least three times. Quantitative data were presented as mean ± standard error of the mean, and analyzed by GraphPad Prism 8.0 software (San Diego, CA, USA). Two-tailed Student's *t*-test, one-way ANOVA, and two-way ANOVA followed by the Student-Newman-Keuls (SNK) test were used for significance calculations, and *P* < 0.05 was considered statistically significant.

## Results

### α-ENaC was expressed in both human and mouse pancreatic β-cells

**Figure 1:**
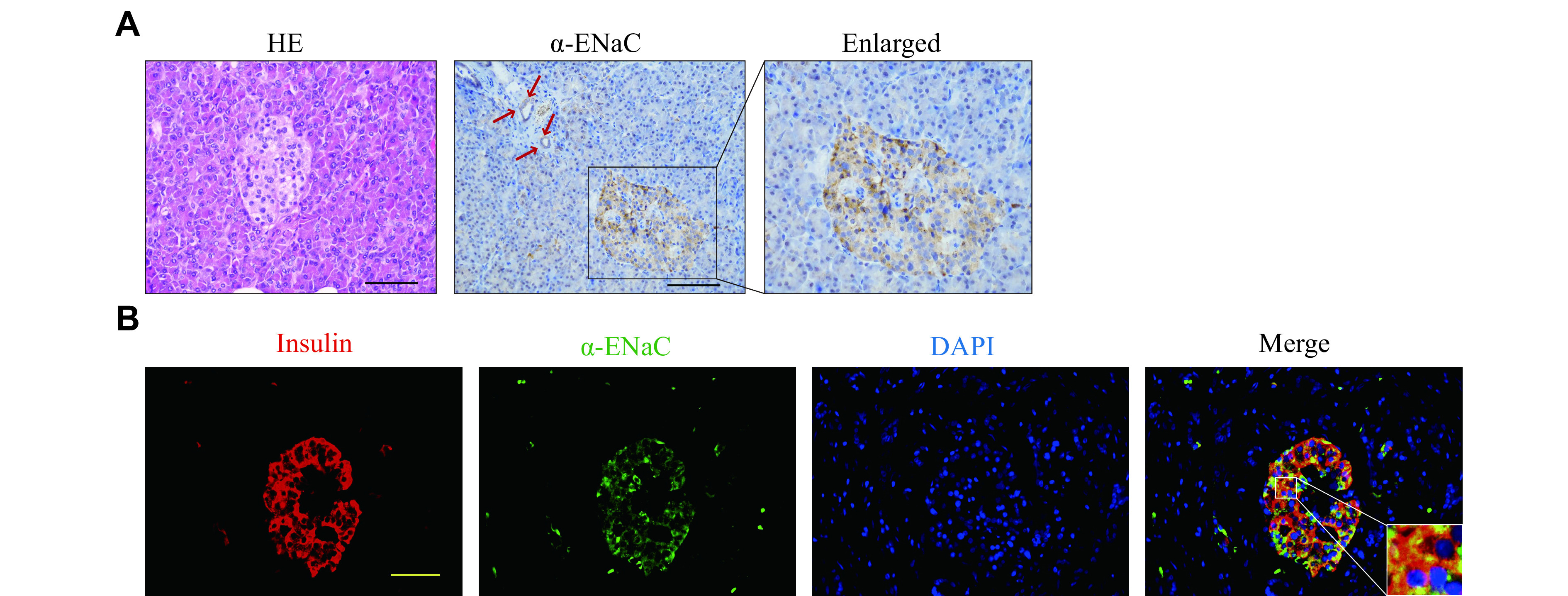
α-ENaC was expressed in human pancreatic β-cells.

**Figure 2:**
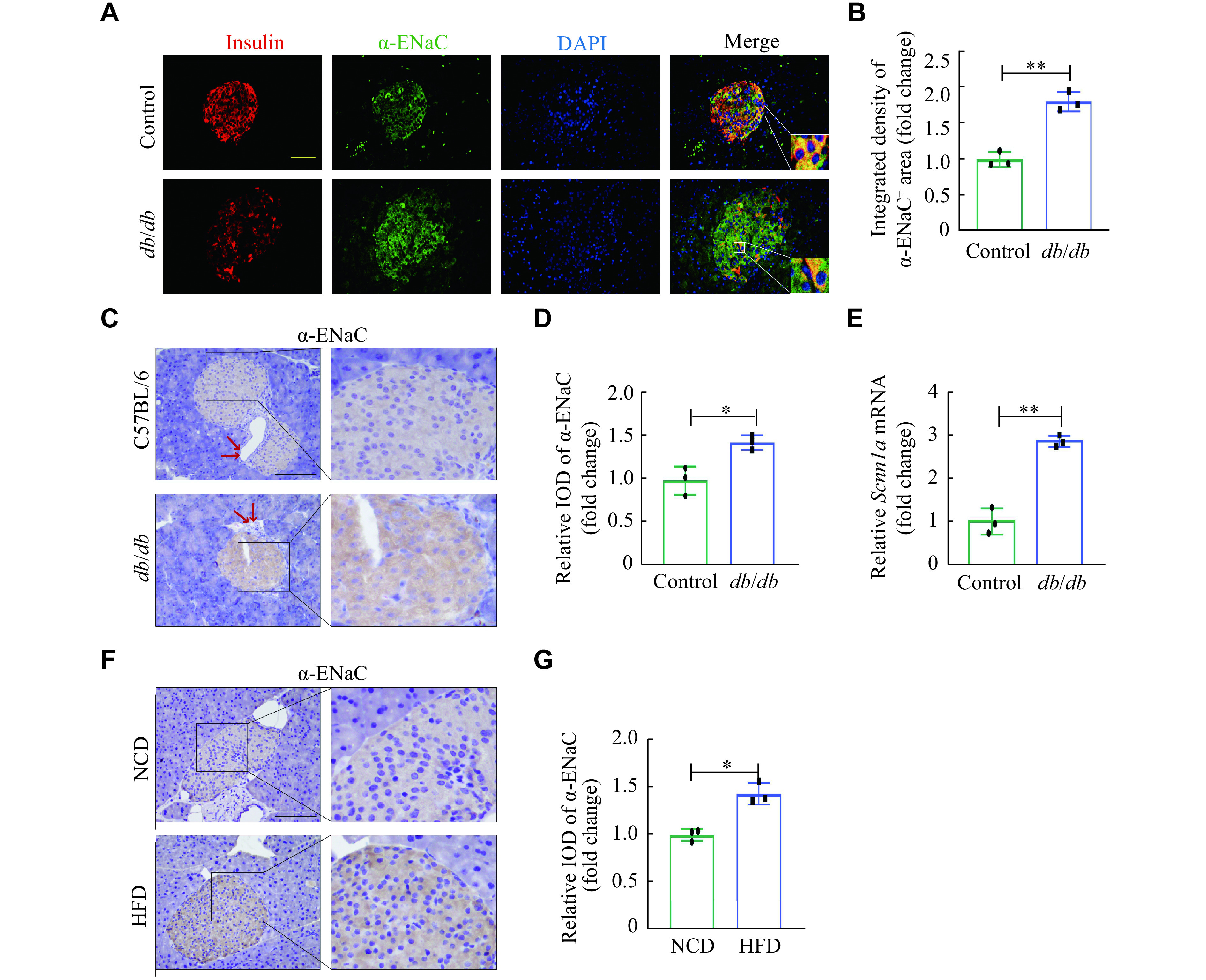
α-ENaC expression was up-regulated in pancreatic β-cells in *db/db* mice and HFD mice.

Initially, we investigated the presence of α-ENaC in the human pancreas. The HE staining revealed a normal histological morphology of the human pancreatic tissues used in the current study, while the IHC staining demonstrated the expression of α-ENaC in the pancreatic glandule and islet of the pancreas (***Fig. 1A***). The IF staining revealed that the human α-ENaC was mainly co-localized with the β-cell marker insulin (***Fig. 1B***), while it also co-localized with the α-cell marker glucagon (***Supplementary Fig. 1A***, available online). The semi-quantitative data showed that the number of β-cells expressing α-ENaC was approximately 82.2% (± 4.2%), which was significantly higher than the number of α-cell expressing α-ENaC (approximately 16.5% [± 4.6%]) (***Supplementary Fig. 1B***, available online). Moreover, the IF data revealed that human α-ENaC localized in the cytoplasm and cell membrane of β-cells (***Fig. 1B*** and ***Supplementary Fig. 1A***). We also found that α-ENaC protein was expressed in mouse β-cells and localized in the cytoplasm and cell membrane (***Fig. 2A***). However, mouse α-ENaC was not expressed in mouse α-cells (***Supplementary Fig. 1C***, available online).

### α-ENaC in the pancreas was upregulated in *db/db* mice and HFD mice

We examined the fasting blood glucose levels in the *db/db* mice and HFD-induced obesity mice. The results showed that the levels of blood glucose were significantly higher in both *db/db* and HFD mice, compared with the control and NCD mice, respectively (***Supplementary Fig. 1D*** and ***1E***, available online), indicating abnormal glucose metabolism in these animal models. The IF staining revealed a significant elevation of α-ENaC in the islets of *db/db* mice, compared with the control mice (***Fig. 2A*** and ***2B***). Consistently, the IHC results demonstrated that the α-ENaC expression level in the islets of *db/db* mice was 1.5-fold higher than that in the control mice (***Fig. 2C*** and ***2D***, *P* < 0.05). Subsequently, the qRT-PCR data showed that the transcription level of α-ENaC (*Scnn1a*) in the *db/db* mice was nearly three-fold higher than that in the control mice (***Fig. 2E***). In the HFD-induced obesity mice, we also revealed that α-ENaC expression levels were significantly elevated in the islet tissues, compared with those in the NCD mice (***Fig. 2F*** and ***2G***).

### Up-regulation of α-ENaC in mouse islet β-cells treated by PA

**Figure 3:**
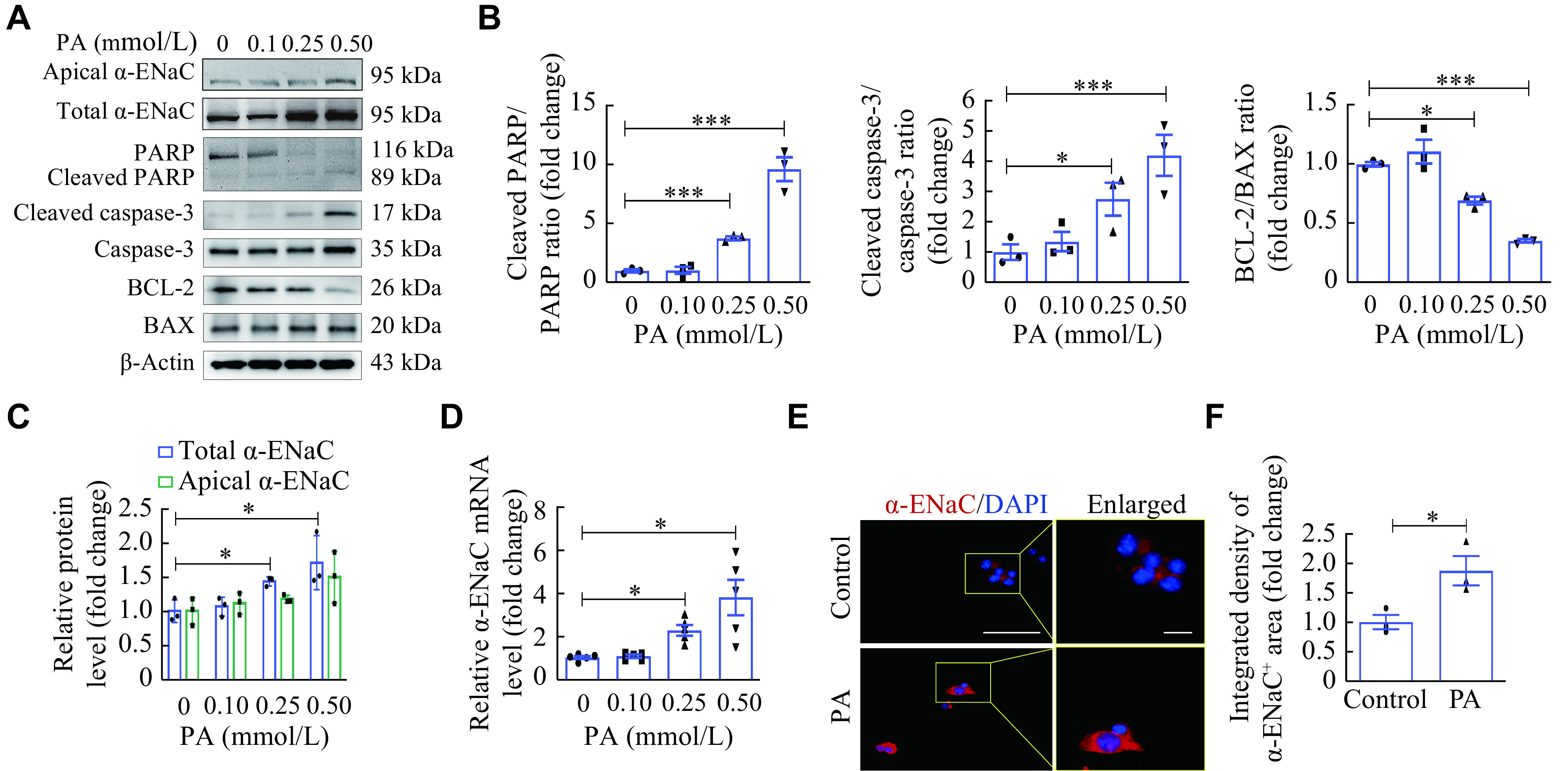
Up-regulation of α-ENaC in mouse islet β-cells treated by palmitate (PA).

Both hyperlipidemia and hyperglycemia are common factors that induce islet injury. To investigate potential involvement of α-ENaC in these processes, we evaluated the expression of α-ENaC in MIN6 cells stimulated with PA for 24 h or high glucose for 48 h. The results revealed that PA induced expression levels of the cleaved PARP, cleaved caspase-3, and BAX, but decreased expression levels of BCL-2 in MIN6 cells, in a dose-dependent manner (***Fig. 3A*** and ***3B***), indicating that PA induced MIN6 cell apoptosis. Consistently, TUNEL (***Supplementary Fig. 2A*** and ***2B***, available online) and flow cytometry data (***Supplementary Fig. 2C***, available online) also showed that PA triggered the apoptosis of MIN6 cells in a dose-dependent manner.

The total α-ENaC protein levels were upregulated in PA-treated MIN6 cells (***Fig. 3A*** and ***3C***), but there were no expression changes of α-ENaC in the glucose-treated MIN6 cells (***Supplementary Fig. 2D*** and ***2E***, available online). Consistently, qRT-PCR and IF staining results also showed that α-ENaC expression was upregulated at both mRNA and protein levels in the PA-treated MIN6 cells (***Fig. 3D***–***3F***). These results indicate that α-ENaC may be involved in the pathogenesis of pancreatic β-cell injury induced by PA.

### α-ENaC participated in insulin synthesis and secretion in β-cells

**Figure 4:**
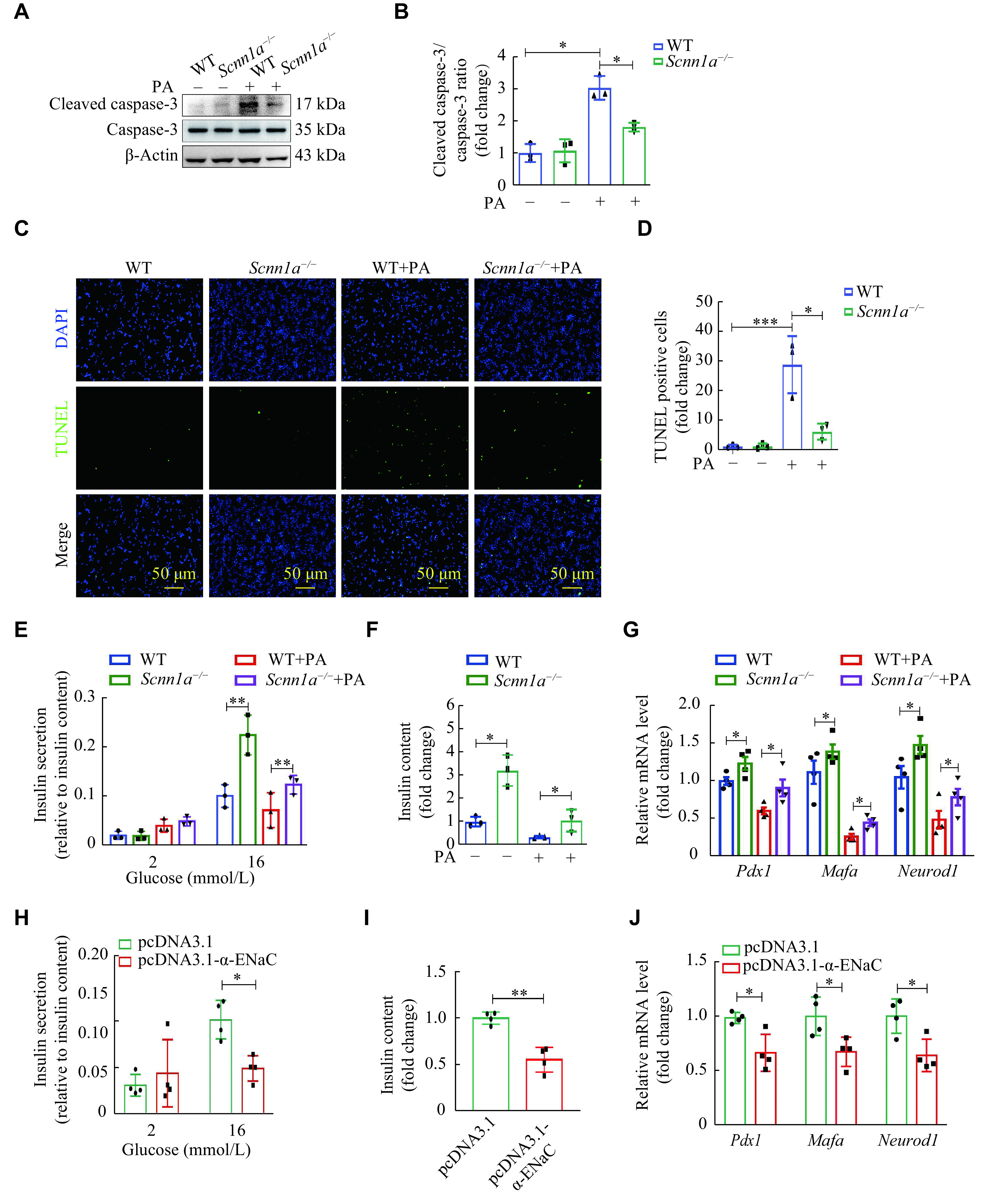
α-ENaC participated in insulin synthesis and secretion in β-cells.

To further investigate the role of α-ENaC in islet β-cells, we generated *Scnn1a*^−/−^ MIN6 cell lines by CRISPR/Cas9^[35]^. The CCK-8 assay was performed to detect the cell viability of wild-type (WT) and *Scnn1a*^−/−^ MIN6 cells stimulated with or without PA. The results showed that PA treatment significantly decreased the viability of MIN6 cells, whereas α-ENaC knockout exhibited a rescuing effect on cellular viability (***Supplementary Fig. 3A***, available online). Mechanistically, the knockout of α-ENaC in MIN6 cells significantly attenuated the PA-induced upregulation of cleaved caspase-3, an established apoptosis marker (***Fig. 4A*** and ***4B***). Consequently, this led to a reduction in the overall apoptotic extent as demonstrated by the results of the TUNEL assay (***Fig. 4C*** and ***4D***). The flow cytometry experiments confirmed the results of the biochemistry and TUNEL assays (***Supplementary Fig. 3B***, available online). In addition, amiloride (an α-ENaC inhibitor) pretreatment showed a similar effect of suppressing the up-regulation of cleaved caspase-3 induced by PA in MIN6 cells (***Supplementary Fig. 3C*** and ***3D***, available online).

We next performed assays of the glucose-stimulated insulin secretion in WT and *Scnn1a*^−/−^ MIN6 cells, stimulated with or without PA. As shown in ***Fig. 4E*** and ***4F***, the insulin content in *Scnn1a*^−/−^ MIN6 cells was higher than that in WT cells (*P* < 0.05), and the insulin secretion in *Scnn1a*^−/−^ MIN6 cells was higher than that in WT cells after glucose stimulation (*P* < 0.01). The PA treatment suppressed the synthesis and secretion of insulin, which was blunted in the α-ENaC knockout condition. The mRNA levels of the insulin transcription factors, such as *Pdx1*, *Mafa*, and *Neurod1*, were higher in the *Scnn1a*^−/−^ cells than those in the WT MIN6 cells with or without PA stimulation (*P* < 0.05) (***Fig. 4G***). These results indicate that the loss of α-ENaC ameliorated the dysfunction induced by PA in β-cells.

To further identify the effects of α-ENaC on the pancreatic β-cell functions, we transfected equal amounts of α-ENaC overexpression plasmid (pcDNA3.1-α-ENaC) or vector plasmid (pcDNA3.1) into MIN6 cells. The α-ENaC overexpression was confirmed by Western blotting assay (***Supplementary Fig. 4A***, available online). Overexpression of α-ENaC in MIN6 cells significantly suppressed the glucose-induced insulin secretion and insulin content (***Fig. 4H*** and ***4I***). The mRNA levels of insulin transcription factors, including *Pdx1*, *Mafa*, and *Neurod1*, exhibited a significant reduction in MIN6 cells with α-ENaC overexpression, compared with the control cells (***Fig. 4J***).

In addition, Western blotting assays revealed that the α-ENaC overexpression further increased the expression of cleaved caspase-3 in MIN6 cells with PA stimulation (***Supplementary Fig. 4A*** and ***4B***, available online). The TUNEL assay also showed that the apoptotic rate of MIN6 cells with α-ENaC overexpression, followed by PA treatment, was significantly higher than those treated with PA alone (normalized to the normal group) (***Supplementary Fig. 4C*** and ***4D***, available online). These data show that α-ENaC participates in insulin synthesis and secretion in β-cells.

### α-ENaC regulated the ubiquitylation and degradation of SIRT2

**Figure 5:**
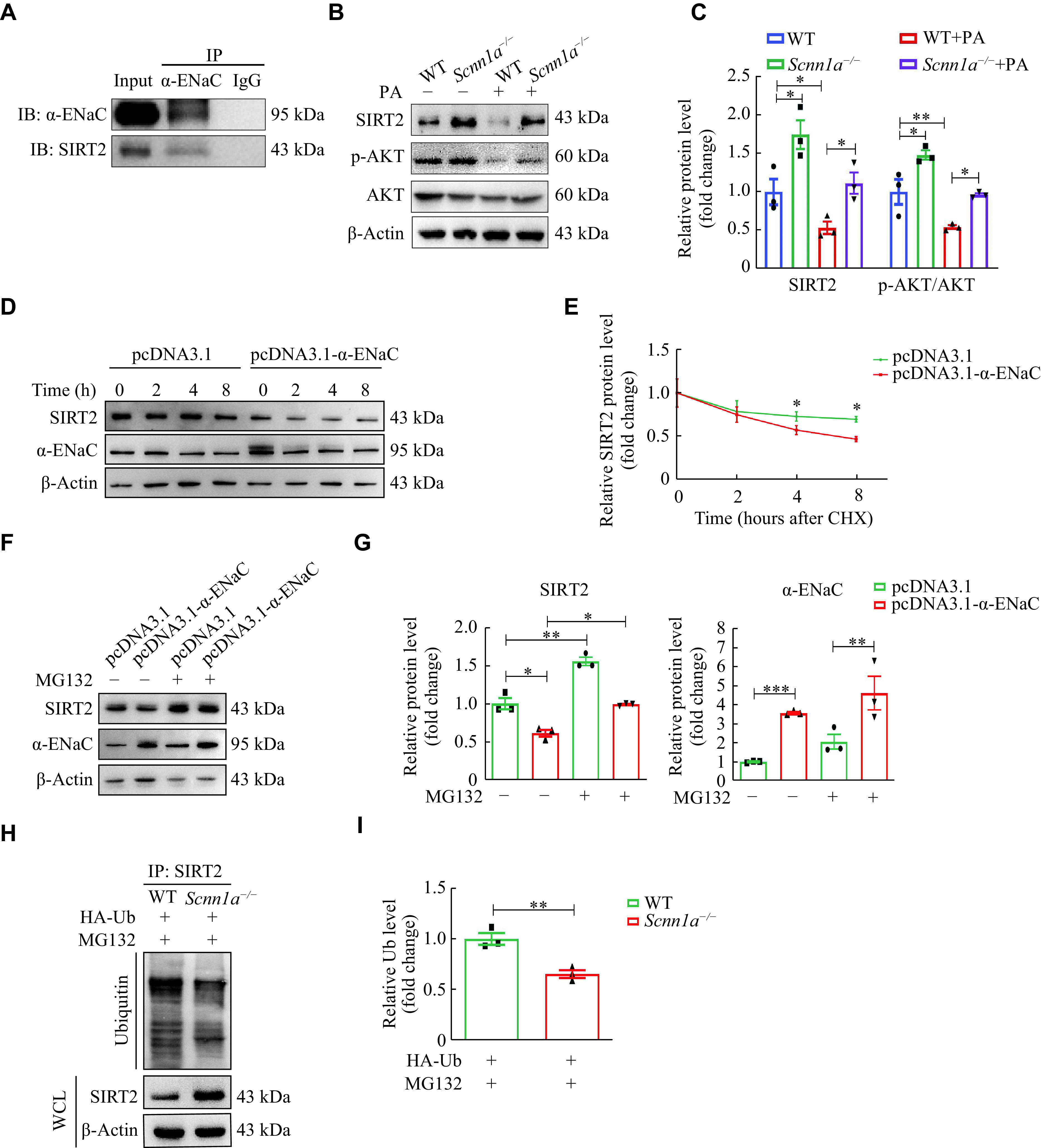
α-ENaC regulated the ubiquitylation and degradation of SIRT2.

To gain insights into the roles of α-ENaC in β-cell dysfunction, liquid chromatography and tandem mass spectrometry (LC-MS/MS) were performed to screen for the α-ENaC binding proteins in MIN6 cells. As shown in ***Table 1***, the peptides identified by LC-MS/MS were categorized based on their functions, including protein binding, enzyme binding, nucleotide binding, hydrolase activity, heat shock proteins, and structural constituent of ribosome. SIRT2 was one of the top α-ENaC binding proteins. The interaction between α-ENaC and SIRT2 was confirmed by Co-immunoprecipitation (Co-IP) assay (***Fig. 5A***). We also conducted the Co-IP assay to detect the interaction between BIP (*Hspa5*) and α-ENaC. However, the binding signal between BIP and α-ENaC was very weak (***Supplementary Fig. 5A***, available online).

**Table 1:** Selected α-ENaC binding proteins in MIN6 cells

Functions	Genes	Proteins	Unique peptides	iBAQ
Protein binding	*Naa50*	Isoform 3 of N-alpha-acetyltransferase 50	5	8426.50
	*Ckap4*	Cytoskeleton-associated protein 4	5	589.14
	*C3*	Complement C3	4	620.93
	*Sgta*	Isoform 2 of Small glutamine-rich tetratricopeptide repeat-containing protein alpha	3	1006.20
Enzyme binding	*Sirt2*	NAD-dependent protein deacetylase sirtuin-2	4	1070.50
	*Ywhaz*	14-3-3 protein zeta/delta	2	227.74
	*Gapdh*	Glyceraldehyde-3-phosphate dehydrogenase	2	5346.50
Nucleotide binding	*Eef1a1*	Elongation factor 1-alpha 1	5	2156.70
	*Tubb4b*	Tubulin beta-4B chain	3	1117.00
	*Abce1*	ATP-binding cassette sub-family E member 1	2	353.32
	Acly	ATP-citrate synthase	2	269.09
Hydrolase activity	*Nt5dc3*	5′-nucleotidase domain-containing protein 3	4	1327.20
Heat shock protein	*Hspa5*	78 kDa glucose-regulated protein	8	2141.70
	*Hspa8*	Heat shock cognate 71 kDa protein	8	1559.50
Structural constituent of ribosome	*Rps3*	40S ribosomal protein S3	3	2948.60
	*Rpl4*	60S ribosomal protein L4	3	906.06
	*Rps6*	40S ribosomal protein S6	3	399.28
	*Rps8*	40S ribosomal protein S8	3	367.98
	*Rpl12*	60S ribosomal protein L12	2	360.30
	*Rpl18*	60S ribosomal protein L18	2	2412.10
	*Rps16*	40S ribosomal protein S16	2	754.59
	*Rps2*	40S ribosomal protein S2	2	1850.90
	*Rps20*	40S ribosomal protein S20	2	2506.00
Abbreviation: iBAQ, intensity-based absolute quantification.				

The Western blotting assays revealed that SIRT2 expression and the activation of its downstream AKT in *Scnn1a*^−/−^ in MIN6 cells were increased, compared with WT MIN6 cells (***Fig. 5B*** and ***5C***). The PA stimulation inhibited the expression of p-AKT/AKT (decreased by 45.9% [± 4.4%], *P* < 0.01) in WT MIN6 cells, while α-ENaC knockout relieved this inhibitory effect of PA to a certain extent (decreased by 35.1% [± 2.1%], *P* < 0.05) (***Fig. 5B*** and ***5C***). Next, we evaluated the effect of α-ENaC on the SIRT2 protein stability by CHX chase assays and found that the half-life of SIRT2 in MIN6 cells was decreased with α-ENaC overexpression (***Fig. 5D*** and ***5E***). These results indicate that α-ENaC may weaken the SIRT2 stability in pancreatic β-cells.

As the SIRT2 protein was reportedly degraded by ubiquitination^[39–40]^, we hypothesized that α-ENaC might regulate SIRT2 protein levels by controlling its ubiquitination status. As shown in ***Fig. 5F*** and ***5G***, α-ENaC overexpression decreased SIRT2 expression, while MG132 (a proteasomal degradation inhibitor) treatment significantly restored SIRT2 protein levels. Consistently, the extent of ubiquitin of SIRT2 was significantly lower in *Scnn1a*^−/−^ MIN6 cells than in WT MIN6 cells (***Fig. 5H*** and ***5I***).

Studies have indicated that SIRT2 plays a protective role by inhibiting ER stress induced by multiple damaging factors, including fatty acids^[41–42]^. In the current study, we found that overexpression of SIRT2 attenuated the upregulation of both XBP1s and CHOP induced by PA in MIN6 cells (***Supplementary Fig. 5C*** and ***5D***, available online), indicating that the decreased SIRT2 expression mediated by α-ENaC may contribute to the promotion of ER stress in the injured MIN6 cells.

### α-ENaC modulated β-cell function related to IRE1α/XBP1 and PERK/CHOP pathways

**Figure 6:**
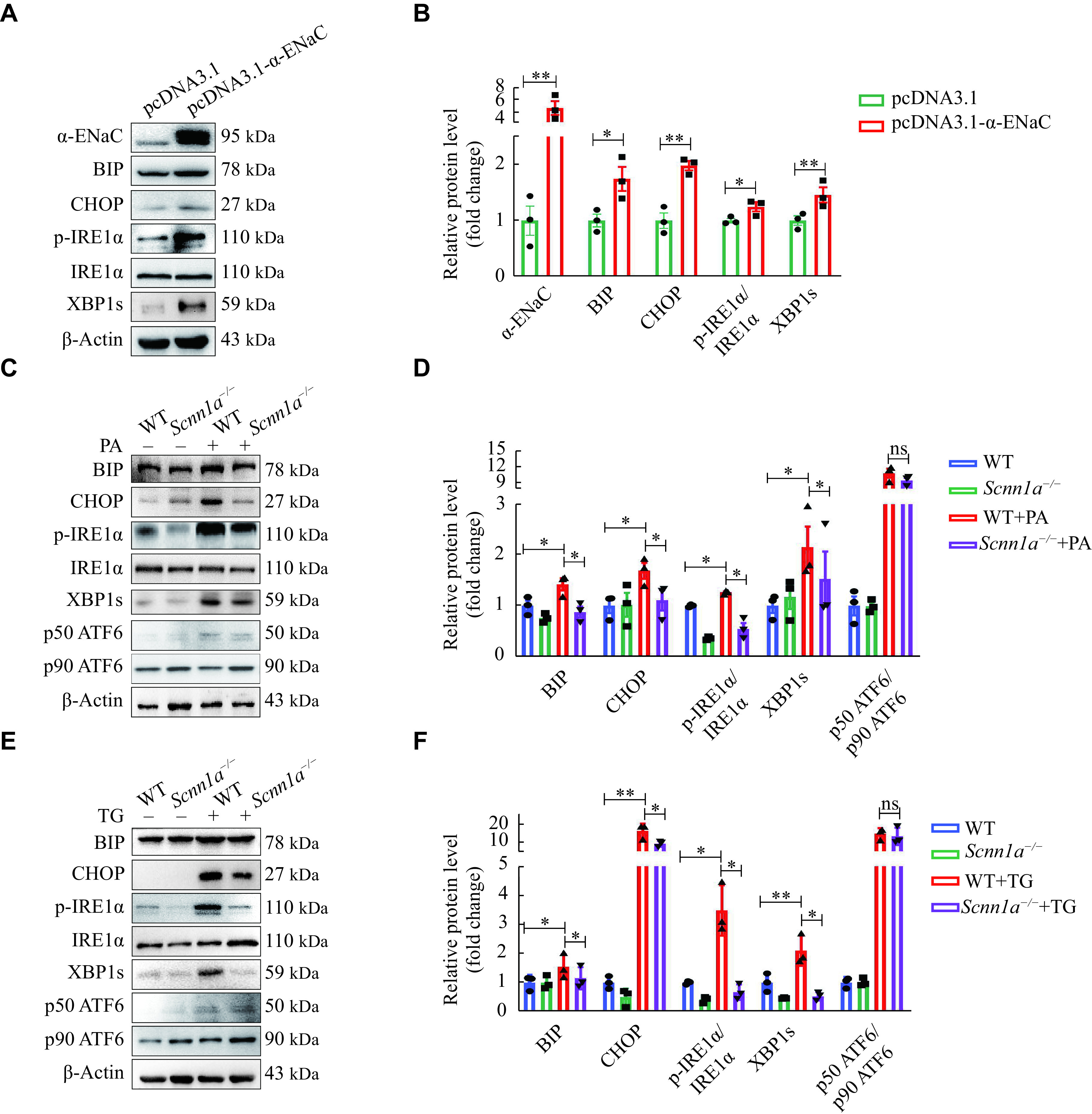
α-ENaC mediated ER stress in pancreatic β-cells.

ER stress plays a role in the pathogenesis of islet disorders and β-cell injury^[43]^. To investigate the effects of α-ENaC on pancreatic β-cell survival and function associated with ER stress, we detected the expression of a series of ER stress markers in MIN6 cells with different expression levels of α-ENaC. As shown in ***Fig. 6A*** and ***6B***, α-ENaC overexpression significantly upregulated the expressions of BIP, CHOP, p-IRE1α, and XBP1s in MIN6 cells. Furthermore, the elevation of BIP, CHOP, p-IRE1α, and XBP1s induced by PA stimulation was attenuated in *Scnn1a*^−/−^ MIN6 cells, compared with that of WT MIN6 cells (***Fig. 6C*** and ***6D***). However, there was no significant change in ATF6 expression between *Scnn1a*^−/−^ and WT MIN6 cells (***Fig. 6C*** and ***6D***).

To investigate the role of α-ENaC in MIN6 cell injury, we treated these cells with 0.1 μmol/L TG, an activator of ER stress, for 24 h. The results showed that TG significantly upregulated the expressions of BIP, CHOP, p-IRE1α, XBP1s, and ATF6, while α-ENaC knockout blunted the TG-induced upregulation of IRE1α/XBP1 markers, but not ATF6 (***Fig. 6E*** and ***6F***). These results suggest that IRE1α/XBP1 and PERK/CHOP pathways, but not the ATF6 pathway, may be involved in the regulation of pancreatic β-cell functions by α-ENaC.

## Discussion

**Figure 7:**
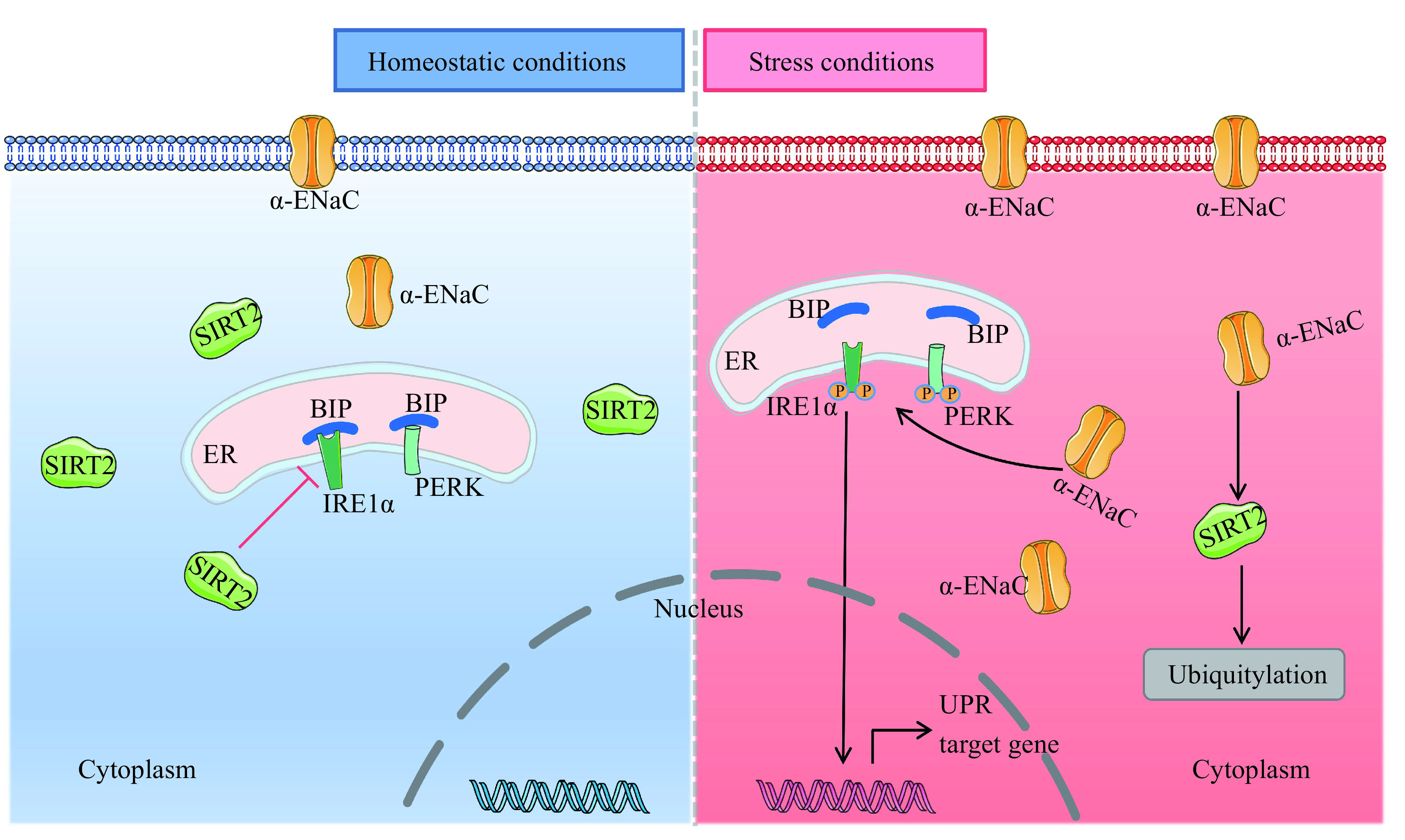
A schematic diagram showing the role of α-ENaC in the dysfunction of pancreatic β-cells.

According to the human protein atlas database (https://www.proteinatlas.org), α-ENaC is expressed in exocrine glandular cells and pancreatic endocrine cells in the human pancreas. Our findings further demonstrated that α-ENaC was expressed in pancreatic glandule and pancreatic cells in the healthy human pancreas. We also discovered that overexpression of α-ENaC in MIN6 cells significantly reduced insulin content and glucose-induced insulin release. In contrast, α-ENaC deficiency in MIN6 cells attenuated the synthesis and secretion of insulin that were both suppressed by PA. Furthermore, we found that α-ENaC regulated the ubiquitylation and degradation of SIRT2 in β-cells. Additionally, α-ENaC modulated islet β-cell function in ways linked to the IRE1α/XBP1 and PERK/CHOP ER stress pathways, supporting potential novel roles of α-ENaC in pancreatic β-cells (***Fig. 7***). As a result, we provided evidence that α-ENaC was correlated with the production and release of insulin in β-cells, and that α-ENaC overexpression enhanced islet β-cell malfunction.

Pancreatic β-cells secrete insulin in response to elevated blood glucose levels and play a significant role in glucose homeostasis. Islet β-cells express multiple ion channels that are involved in insulin secretion, including K_ATP_, Na_v_, and Ca_v_. However, the plasma membrane of β-cells may contain additional, undiscovered ion channels^[10–12]^. One study found that sodium glucose co-transport 2 (SGLT2) inhibitor restored insulin content and β-cell function by reducing oxidative and ER stress of β-cells in monogenic neonatal diabetes mellitus with insulin secretory-deficient^[44]^, which showed that SGLT was involved in the control of β-cell function. In our previous reports, we showed that ENaC, a voltage-independent sodium ion channel, was correlated with β-cell function^[35]^, and we investigated the underlying mechanism for the first time. It is interesting that we also observed the increased expression levels of α-ENaC in islets of mice with T2DM in the current study, which may be present in some dedifferentiated or transdifferentiated cells. Because more data are needed to support this, we will continue to study in depth in the future.

Traditionally, the basic function of ENaC is thought to be as a sodium channel localized in the cell membrane; however, with further ENaC research in recent years, it has been found that ENaC specifically combines with some proteins in the cytoplasm to play a biological function and affects the development of diseases^[26,30,45–46]^. In the current study, we revealed that the total α-ENaC expression was increased in β-cells under the injury conditions, but not at an apical α-ENaC level. These findings suggest that α-ENaC plays a novel function in the state of β-cell injury. Hence, we hypothesize that intracellular α-ENaC may be involved in the process of islet β-cell injury through direct or indirect pathways. It has been reported that the transcription level of α-ENaC is regulated by FOXO1, ERK kinase-signaling pathway, and mTOR-related pathway^[47–49]^, however, future studies are needed to clarify the regulatory mechanism of α-ENaC in islet β-cells. ER stress is considered one of the underlying causes of T2DM^[50]^, and a large number of studies have proved that the long-term enhanced ER stress causes cell apoptosis and leads to dysfunction of the islet cell by reducing the expression of key transcription factors like PDX1 and MAFA, which are involved in regulating insulin transcription^[51–54]^. SCNN1B, which encodes the β-subunit of ENaC, induces cell apoptosis by promoting the production of the ER stress-related proteins^[30]^. Moreover, the endoplasmic reticulum chaperone 170 (GRP170) is involved in the degradation and assembly of α-ENaC, while the activation of ER stress is detected in the kidney tissue of *Grp170*-specific knockout mice^[55–56]^. All these data point to a potential role for α-ENaC in the ER stress pathway, albeit additional precise investigations need to be done.

Moderate ER stress improves intracellular protein folding ability and inhibits protein production, which is beneficial to restore ER homeostasis. However, a long-term activation of ER stress causes apoptosis and cell damage^[57–58]^. The unfolded protein response is the main pathway activated by ER stress and is regulated by three signaling pathways: IRE1α/XBP1, ATF6, and PERK/CHOP^[59–60]^. Our current findings suggest that α-ENaC affects the occurrence of ER stress and is involved in the process of islet β-cell damage. Furthermore, we examined all three signaling pathways and found that the change in the expression of α-ENaC did not affect the activation of ATF6 (the expression of p50 ATF6), so it is likely that α-ENaC is involved in regulating the IRE1α/XBP1 and PERK/CHOP pathways, but not the ATF6 signaling pathway, ultimately affecting islet β-cell survival and function.

SIRT2 is a member of the sirtuin family that includes NAD^+^-dependent class Ⅲ protein deacetylases, and is involved in the AKT /GSK-3β pathway to support pancreatic β-cell function^[37,61]^. Numerous investigations have demonstrated that pharmacological inhibition of SIRT2 or the *SIRT2* gene deletion inhibits insulin secretion^[62–63]^. We also showed in the current study that α-ENaC contributed to SIRT2 degradation. However, as a functional protein, α-ENaC may indirectly influence the degradation of SIRT2, but it is unclear which element directly affects SIRT2 in islet β-cells. Therefore, understanding the effect of α-ENaC on SIRT2 degradation still needs to be studied in the future.

In summary, our findings demonstrate pathogenic significance of excessive α-ENaC in β-cell dysfunction and indicate the necessity of α-ENaC-targeting therapies to prevent SIRT2 loss and inhibit T2DM development. Unfortunately, we were not able to study the expression and function of α-ENaC in diabetic patients, and more investigation is needed to fully understand the effect of ENaC on β-cells.

## SUPPLEMENTARY DATA

Supplementary data to this article can be found online.
