# Supplementary material for: Upregulation of α-ENaC induces pancreatic β-cell dysfunction, ER stress, and SIRT2 degradation
Source: J Biomed Res. 2024 May 21;38(3):241–55. doi: 10.7555/JBR.37.20230128 (PMC11144933; doi:10.7555/JBR.37.20230128)
Supplement: Supplementary file 1 — Supplementary data to this article can be found online. [file jbr-38-3-241-S1.pdf]

Available online at www.jbr-pub.org.cn

Open Access at PubMed Central

Journal of Biomedical Research, 2024 0(000): 1–5

Supplementary Data

JBR

Excessive α-ENaC promotes β-cell dysfunction

Xue Zhang[1,4,△], Dan Zhang[1,4,△], Lei Huo[1], Xin Zhou[1], Jia Zhang[2], Min Li[3], Dongming Su[3], Peng Sun[2], Fang Chen[2,✉], Xiubin Liang[1,✉]

[1]*Department of Pathophysiology, Nanjing Medical University, Nanjing, Jiangsu 211166, China;*
[2]*Key Laboratory of Human Functional Genomics of Jiangsu Province, Department of Biochemistry and Molecular Biology, Nanjing Medical University, Nanjing, Jiangsu 211166, China;*
[3]*Department of Pathology, Nanjing Medical University, Nanjing, Jiangsu 211166, China;*
[4]*Department of Pathology, Nanjing Drum Tower Hospital, the Affiliated Hospital of Nanjing University Medical School, Nanjing, Jiangsu 210009, China.*

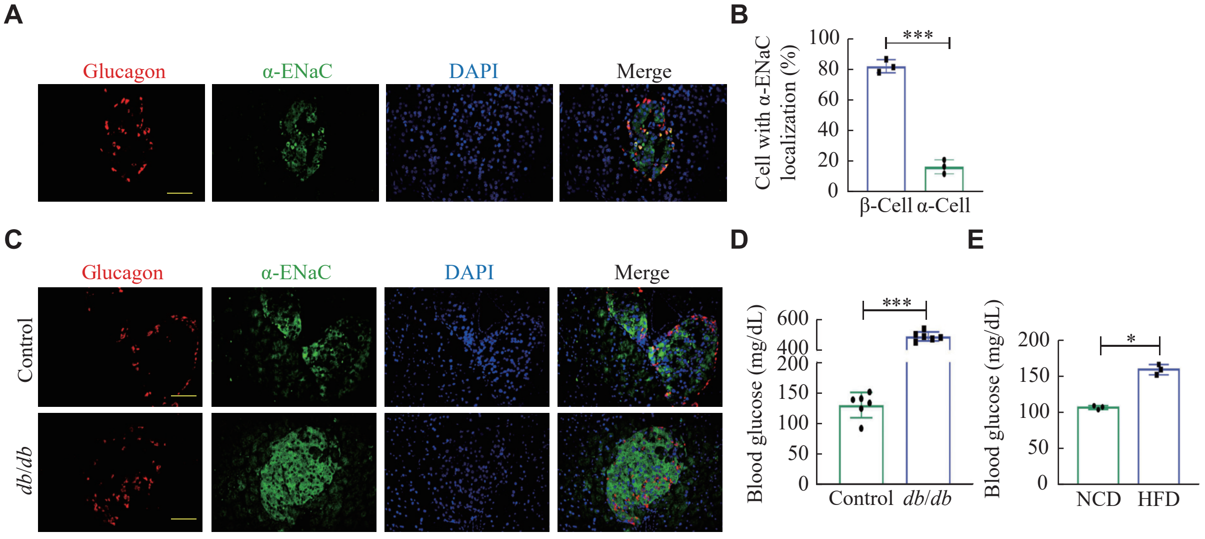

Supplementary Fig. 1 **Expression of α-ENaC in islet β-cells.** A: Immunofluorescence staining of glucagon and α-ENaC in the human pancreas tissues (n = 5). B: Quantitative analysis of α-ENaC (green) colocalizing with β-cells (insulin) in *Fig. 1B* and α cells (glucagon) in panel A. C: Immunofluorescence staining of glucagon and α-ENaC in the pancreatic tissues of control and *db*/*db* mice. D: Fasting blood glucose levels of *db*/*db* mice and control mice (n = 6). E: Fasting blood glucose levels of HFD mice and NCD mice (n = 3). Data are presented as means ± standard error of the mean, $^{*}P < 0.05$ and $^{***}P < 0.01$ (two-tailed Student's t-test). Scale bar: 100 µm. Abbreviations: NCD, normal chow diet; HFD, high-fat diet.

△These authors contributed equally to this work.

✉Corresponding authors: Fang Chen, Department of Biochemistry and Molecular Biology, Nanjing Medical University, 101 Longmian Avenue, Nanjing, Jiangsu 211166, China. E-mail: chenfang@njmu.edu.cn; Xiubin Liang, Department of Pathophysiology, Nanjing Medical University, 101 Longmian Avenue, Nanjing, Jiangsu 211166, China. E-mail: liangxiubin@njmu.edu.cn.

Received: 22 May 2023; Revised: 05 November 2023; Accepted: 11 November 2023; Published online: 21 May 2024

CLC number: R587.1, Document code: A

The authors reported no conflict of interests.

This is an open access article under the Creative Commons Attribution (CC BY 4.0) license, which permits others to distribute, remix, adapt and build upon this work, for commercial use, provided the original work is properly cited.

© 2024 by Journal of Biomedical Research.

https://doi.org/10.7555/JBR.37.20230128

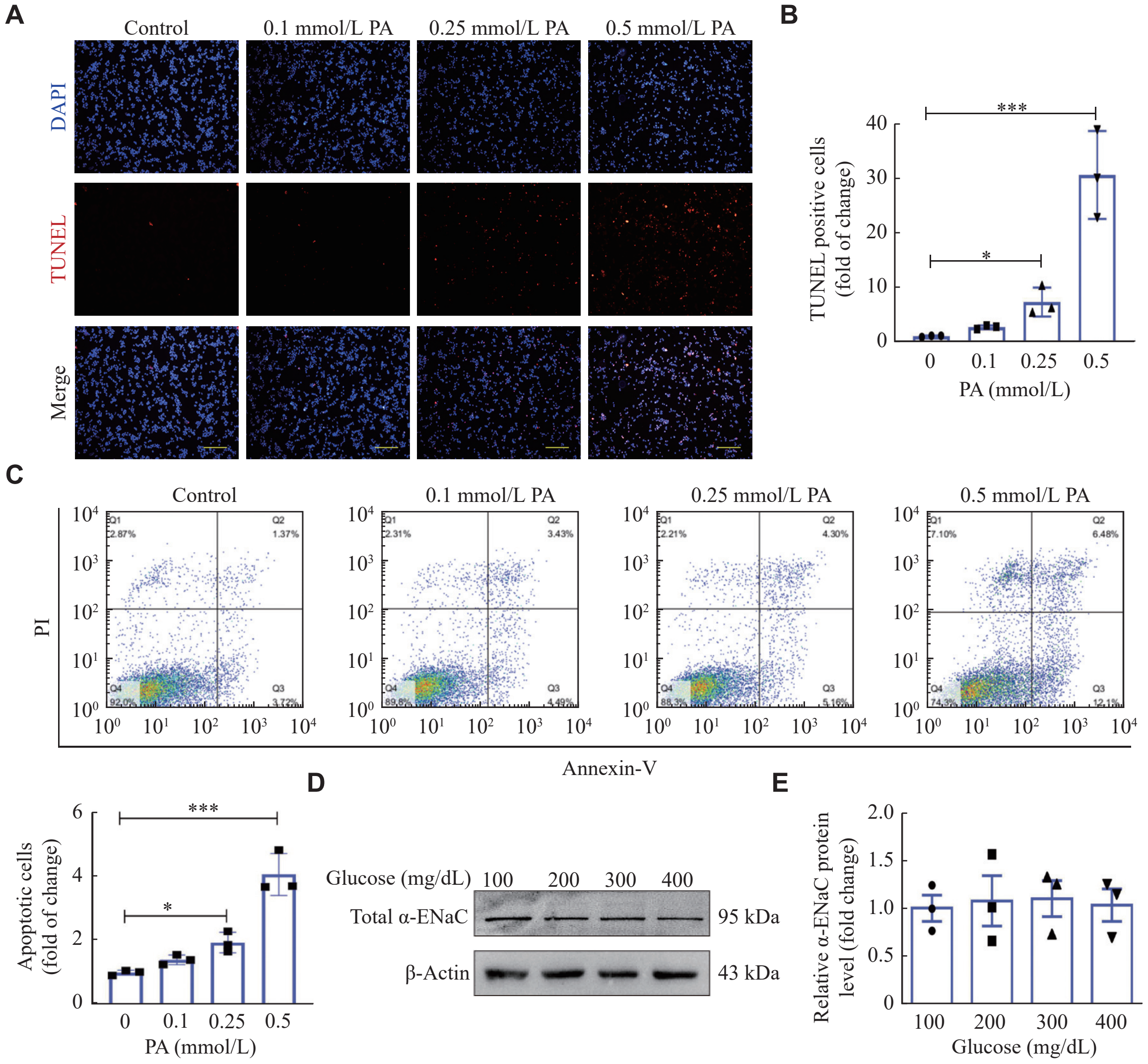

Supplementary Fig. 2 **Detection of apoptosis in MIN6 cells treated by PA.** A: TUNEL staining in MIN6 cells treated with PA. B: TUNEL-positive cells in MIN6 cells treated with PA. C: Annexin-V/PI double fluorescence staining and flow cytometry analysis in MIN6 cells treated with PA. D: Western blotting assay of α-ENaC in MIN6 cells treated with glucose for 24 h. E: Quantitation of the Western blotting data in panel D. Data are presented as means ± standard error of the mean (n = 3). Scale bar: 50 μm. $^{*}P < 0.05$, $^{**}P < 0.01$, $^{***}P < 0.001$ (two-tailed Student's t-test). Abbreviations: PA, palmitate; PI, propidium iodide; Glu, glucose.

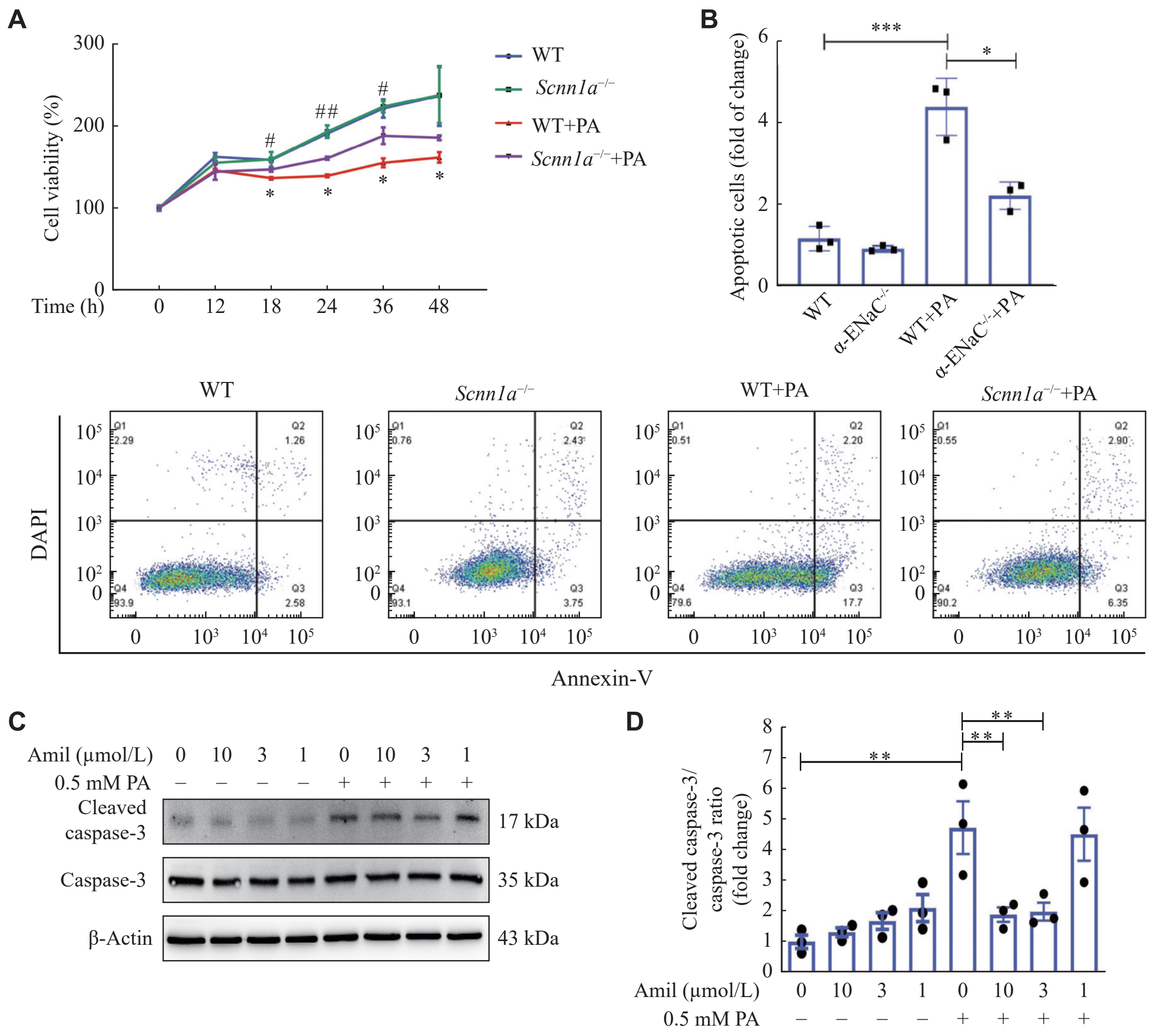

Supplementary Fig. 3 **Knockout or inhibition of α-ENaC ameliorated PA-induced cell damage.** A: The cell viability analysis of the wild-type (WT) and α-ENaC knockout (*Scnn1a*$^{-/-}$) MIN6 cells treated with or without PA. $^{*}P < 0.05$, compared with the WT group; $^{\#}P < 0.05$, and $^{\#\#}P < 0.01$, compared with the WT + PA group. B: Annexin-V/PI double fluorescence staining and flow cytometry analysis of the WT and *Scnn1a*$^{-/-}$ MIN6 cells treated with or without 0.25 mmol/L PA. C: Western blotting assay of cleaved caspase-3 and caspase-3 in MIN6 cells treated with amiloride and PA. D: Quantitation of the Western blotting data in panel C. Data are presented as means ± standard error of the mean ($n = 3$), $^{*}P < 0.05$, and $^{**}P < 0.01$ (two-tailed Student's t-test). Abbreviations: PA, palmitate; WT: wild type; Amil: amiloride.

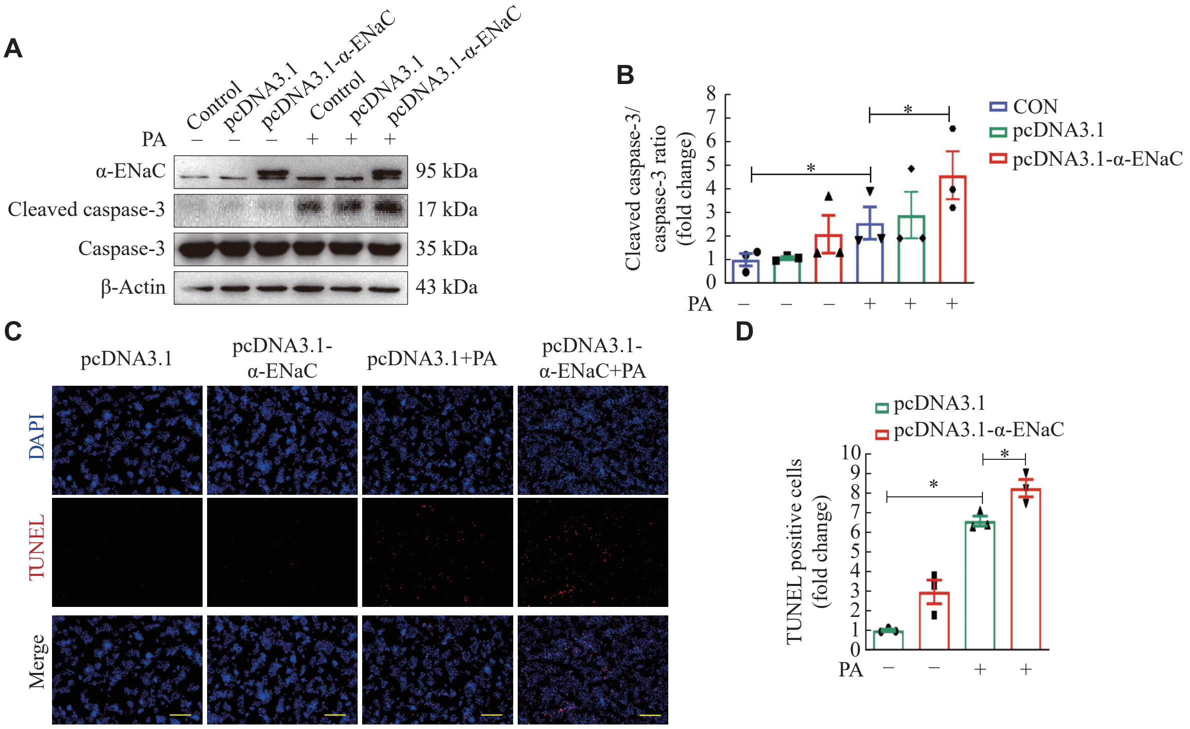

Supplementary Fig. 4 **α-ENaC over-expression further injured MIN6 cells under the PA condition.** A: Western blotting assay of cleaved caspase-3 and caspase-3 in α-ENaC overexpressed MIN6 cells treated with PA. B: Quantification of the Western blotting data in panel A. C: TUNEL staining in α-ENaC overexpressed MIN6 cells treated with PA. D: TUNEL positive cells in α-ENaC overexpressed MIN6 cells treated with PA. Data are presented as means ± standard error of the mean (n = 3). Scale bar: 50 µm, $^{*}P < 0.05$, and $^{**}P < 0.01$ (two-tailed Student's t-test). Abbreviation: PA, palmitate.

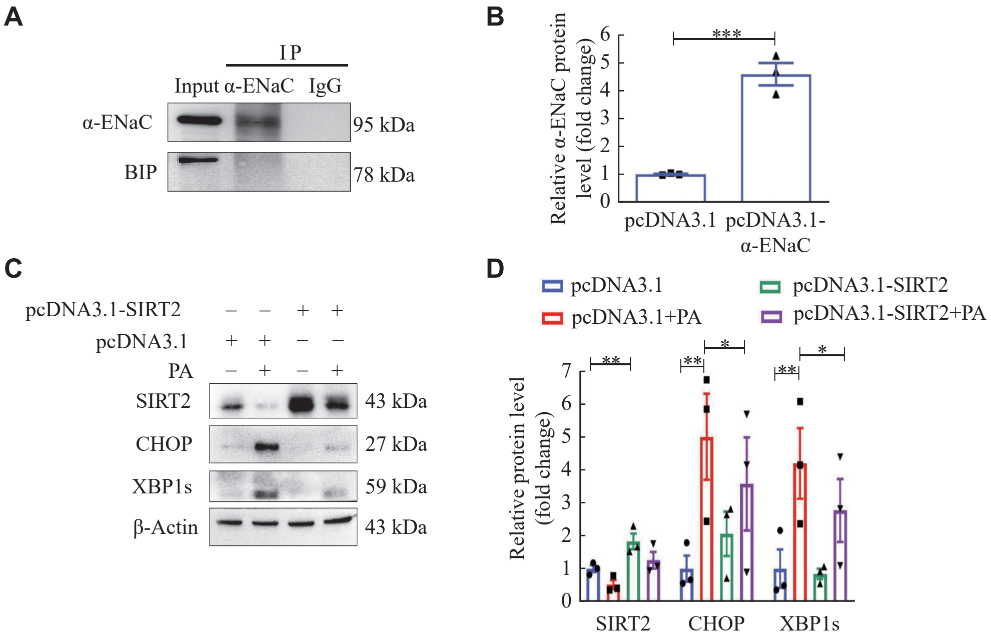

Supplementary Fig. 5 **SIRT2 over-expression attenuated the upregulation of both XBP1s and CHOP induced by palmitate (PA) in MIN6 cells.** A: Co-immunoprecipitation of BIP and α-ENaC in MIN6 cells. B: Quantitation of the α-ENaC expression at time 0 point in ***Fig. 5D***. C: Western blotting assay of XBP1s and CHOP in SIRT2 overexpressed MIN6 cells treated with PA. D: Quantitation of the Western blotting data in panel C. Data are presented as mean ± standard error of the mean (n = 3). $^{*}P < 0.05$ and $^{**}P < 0.01$ by two-tailed Student's t-test.

Supplementary Table 1 **Characteristics of study participants**

Case	Age (years)	Sex	Glucose (mmol/L)	Blood pressure (mmHg)
1	69	Female	4.6	130/70
2	56	Female	5.1	123/85
3	60	Female	4.8	130/75
4	51	Male	5.3	137/65
5	68	Male	4.2	125/80
